# Relationship between Anemia and Falls among Postmenopausal Women in Korea

**DOI:** 10.3390/ijerph19148242

**Published:** 2022-07-06

**Authors:** Yunmi Kim, Jiyun Kim

**Affiliations:** 1 College of Nursing, Eulji University, Seongnam 13135, Korea; kyunm@eulji.ac.kr; 2 School of Nursing, Gachon University, Incheon 21936, Korea

**Keywords:** fall, anemia, energy intake, proteins, dietary iron, vitamin C

## Abstract

This study was conducted to explore the relationship between anemia and falls in postmenopausal women. The relationships between energy, protein, iron, and vitamin C intake were also checked. The data of this study are a combination of data from the Korea National Health and Nutrition Examination Survey (KNHANES) collected from the KNHANES VI-2,3 (2014–2015) and VII-1,2,3 (2016–2018). Falls and anemia were compared according to the subjects’ characteristics. Phi and Cramer’s V coefficient were applied to find the strength of association. Energy, protein, iron, and vitamin C intake and anemia were investigated. The relationship between the status as anemic and the occurrence of falls was analyzed using binary logistic regression. Of the 6665 subjects, 274 had a fall, and 596 had anemia. If the energy intake did not meet the estimated energy requirements, the state of anemia was significantly higher with a weak association. When protein, iron, and vitamin C did not meet the recommended nutrient intake, the anemia state was considerably higher with a weak to moderate association. Postmenopausal women with anemia were more likely to fall than subjects without anemia (OR = 1.88, *p* = 0.003). Additionally, anemia was confirmed to be related to insufficient energy, protein, iron, and vitamin C intake.

## 1. Introduction

Anemia is defined as a blood hematocrit concentration of less than 12.0 g/dL in women [1]. The prevalence of anemia among elderly women in Korea is known to be 12.2% in those 65–74 years old and 20% in the 75–84-year-old population [2]. Among American women in the same age group, the prevalence of anemia among women aged 65–74 is 8.5%, and the prevalence of anemia among women aged 75–84 is 10.3% [3]. In Japan, the prevalence of anemia is 6.4% among women aged 70–74 years and 11.9% among women aged 80–84 years [4]. Anemia tends to increase with age, and the prevalence of anemia among the elderly in Korea is higher than in the United States [5] or Japan [4]. Anemia in the elderly can be viewed as a process of aging [3], but anemia in elderly females is related to inadequate nutrient intake [6]. Anemia seen in postmenopausal women includes iron deficiency anemia and vitamin B12 deficiency anemia [7]. Among these nutrients, vitamin C [8,9,10] and protein are frequently mentioned [10]. Intakes of energy are also related to anemic status [6,10]. Menopause is a process in which a woman’s reproductive function ends with the onset of amenorrhea for 12 months [11]. Menopause is a transition process, and in addition to the deterioration of reproductive function, various changes such as increases in depression and anxiety, decreases in urinary function, sleep disturbances, decreases in physical function, and decreases in bone mineral density occur [12]. Therefore, this period is an important period to adopt healthy behavior and establish a disease prevention strategy [12]. Understanding the etiology of anemia in older women, particularly postmenopausal women, is essential to improving the quality of life and reducing morbidity and mortality in this population, and should thus be a public health priority [13].

Anemia due to iron deficiency is an important health problem in old age as it is related to injuries due to falls [6]. Anemia in the elderly has been confirmed to be related to falls [14] and an adequate assessment of the cause of anemia is necessary [15]. The incidence of falls in elderly females is associated with a decrease in activities of daily living [16], a reduction in social participation [17], and a decrease in quality of life [18], and it is necessary to control fall-related factors in the aging process. The annual amount of direct damage from falls is KRW 150,627, and 666 subjects who experienced falls spent an average medical cost of KRW 455,047, as of 2012 [19]. The incidence of falls is higher in women than in men [20], and the incidence increases with age [21]. Therefore, it is necessary to study factors related to the occurrence of falls in elderly women, and it is necessary to understand the pattern of anemia and its relationship.

Factors related to falls include age, education level, family composition, residential area, economic activity, income level, alcohol consumption, smoking, physical activity, chronic disease, and BMI. In the elderly in the community, the incidence of falls is higher with increasing age [22,23,24,25]. Women have been found to have a higher risk of falls than men [21,22], and the unemployed and blue-collared workers with lower income are at a greater risk of falls [21]. As a result of comparing the fall characteristics of the elderly in rural and urban areas, it has been confirmed that the lifetime fall history of the elderly in rural areas is greater than in urban areas [24]. The lower the educational level, the higher the risk of falls, and the higher the number of falls when living alone than when living with a spouse [21,23,25]. Drinking, smoking, physical activity, and chronic diseases are also associated with falls [25]. As a result of confirming the relationship between BMI and falls, it has been confirmed that the risk of falling is increased when a subject is underweight or overweight, compared to average body weight [26].

In Japan, the prevalence of anemia was investigated, and the relationship between it and falls was studied [14]. However, the relationship between anemia and falls has not been studied much in Korea. It is necessary to investigate whether anemia in postmenopausal women is related to falls to identify the pattern of falls in women as they go through aging from middle age to old age, and whether early intervention strategies are needed to prevent falls. In addition, it is necessary to check which nutrient deficiency is associated with anemia in menopausal women and confirm the relationship between energy, iron, protein, vitamin B12, and vitamin C, which are frequently mentioned. This study is performed to determine whether anemia in postmenopausal women is significantly related to the prevalence of falls by using representative data from Korea.

## 2. Materials and Methods

### 2.1. Data and Study Population

The data of this study are a combination of data from the Korea National Health and Nutrition Examination Survey (KNHANES) collected from the KNHANES VI-2,3 (2014–2015) and VII-1,2,3 (2016–2018). KNHANES is an investigation approved by the Korea Centers for Disease Control and Prevention Institutional Review Board. The review numbers include data from 2014 (2013-12EXP-03-5C) and 2018 (2018-01-03-P-A). In 2015, 2016, and 2017, the KNHANES was carried out without review by the Research Ethics Deliberation Committee as it was conducted by the government for public welfare in accordance with Article 2, Paragraph 1 of the Bioethics Act and Article 2, Paragraph 2 and Paragraph 1 of the Enforcement Regulation of the same Act [27,28]. All participants were informed of the questionnaire by the KNHANES and provided written informed consent.

The KNHANES, comprising national representative data, consists of three components: a health interview, a medical examination, and a nutrition survey [29]. The KNHANES’ sampling plan follows a multi-stage clustered probability design [30]. The health interview and health checkup are conducted by trained staff such as nurses, medical technicians, and health interviewers at the mobile checkup center, while the dieticians visit the home of the participants and conduct a survey on dietary intake [29]. The medical examination includes a collection of human specimens such as urine and blood [30].

**Figure 1:**
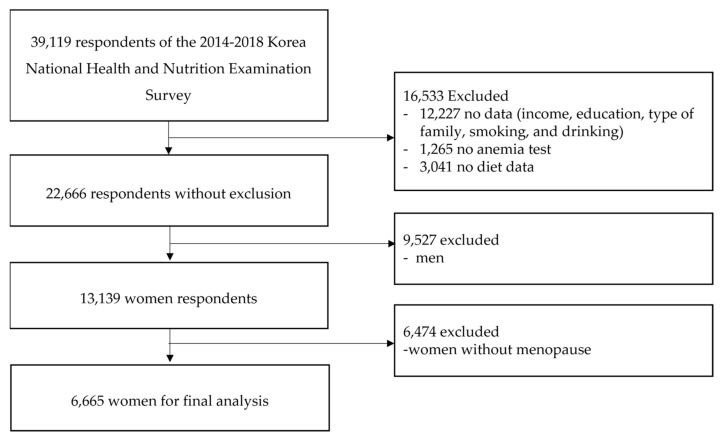
Selection of study subjects.

The selection process of the study subjects for data analysis is shown in Figure 1. A total of 39,199 subjects participated in the National Health and Nutrition Examination Survey from 2014 to 2018; however, 12,227 were excluded due to a lack of information on income, education, type of family, drinking, and smoking. Additionally, 1265 people who did not take an anemia test and 3041 people who did not respond to dietary intake were excluded. Among them, 9527 men were excluded, and 6474 non-menopausal women were excluded out of 13,139 women, and the final data of 6665 women were analyzed. Finally, the women included in the study ranged from 36 to 79 years old. The proportion of artificial menopause was 13.2% of total subjects.

### 2.2. Measurement

#### 2.2.1. Fall

The question “Has there ever been a fall/slip that required treatment at a hospital or emergency room during the past year?” was used to measure whether a fall occurred. If the answer was yes, then we categorized falls as having occurred [28].

#### 2.2.2. Anemia

Anemia in women was defined as a hemoglobin (Hb) level below 12 g/dL according to the World Health Organization (WHO) [1]. Blood sampling for diagnosing anemia is carried out on subjects who have consented to blood collection as part of the health examination and is performed by full-time trained staff [30]. Hemoglobin (Hb) was measured using the sodium lauryl sulfate hemoglobin detection method with XN-9000 (Sysmex, Kobe, Japan) [27].

#### 2.2.3. Nutrient Intake

Nutrient intake was measured based on the nutritional intake questionnaire in the KNHANES. A survey related to the amount of dietary intake was conducted as a 24 h recall survey [27]. To collect nutrient intake information, a dietician visits the subject’s home and conducts a survey using a dish-based, semi-quantitative food frequency questionnaire (FFQ) developed to suit the characteristics of Koreans [30]. The survey items are meal information and food intake information, and a volume calculation tool is used to investigate intake [27]. Daily nutrient intakes were calculated with the coded FFQ data including daily frequencies [31]. For the percentage of the population who consumed less than the estimated energy requirements (EER) and recommended nutrient intake (RNI) for intakes of energy, protein, iron, and vitamin C, data from the 2020 Dietary Reference Intakes for Koreans were referred to [32]. Energy was categorized as to whether the estimated average requirement was met. It was calculated from the median daily nutrient needs of healthy people. In the case of energy, it is assumed that energy is in equilibrium, and the amount of energy required is estimated through energy consumption. Therefore, energy levels were classified by whether they met the EER instead of the term’s average demand. Protein, iron, and vitamin C were classified according to whether the recommended intake amount (RNI) was met. In the case of protein, the daily recommended amount for women is 50 g/day. Iron is 8 g/day for those under 74 and 7 g/day for those over 75. The recommended intake of vitamin C is 100 mg/day [32].

#### 2.2.4. Covariates

The characteristics of the subjects are as follows. Age was classified into less than 64 years old, 65 years old to less than 70 years old, 70 years old to less than 75 years old, and 75 years old to less than 80 years old. Educational level was divided into middle school graduation or lower, high school graduation, and college graduation. The type of family was divided into living alone or not. The residential area was divided into urban and rural areas. Whether or not the person was engaged in economic activity for income was classified. Household income was divided into the top 50% and the bottom 50%. High-risk drinking was defined as drinking 2 to 3 times or more per week and drinking 7 or more drinks per day for men and 5 or more drinks for women [27]. Present smokers were classified by their current smoking status. Aerobic physical activity was classified according to whether a person had practiced moderate-intensity physical activity for 2 h and 30 min or more or 1 h 15 min or more for high-intensity physical activity during the past week [27]. The number of days of high-intensity physical activity, the number of days of moderate physical activity, and the amount of physical activity per day was asked through questionnaires concerning the past week [27]. Physical activity could combine moderate-intensity and high-intensity physical activity with an amount of time equivalent to each activity number [27]. For example, one minute of high intensity equalled 2 min of moderate intensity [27]. Having chronic disease was categorized as being diagnosed with a disorder the interviewee was suffering from or taking medication for. Diseases included hypertension, dyslipidemia, stroke, myocardial infarction or angina pectoris, thyroid disease, osteoarthritis, rheumatoid arthritis, osteoporosis, pulmonary tuberculosis, asthma, diabetes, various cancers, cataracts, glaucoma, macular degeneration, hepatitis B, and hepatitis C, including cirrhosis. BMI was calculated using height and weight and categorized as less than 18.5, 18.5 to 24.9, or 25 or more.

### 2.3. Statistical Analysis

Since the KNHANES data were extracted from complex stratified samples throughout Korea nationwide, the integrated weights proposed in the data analysis guidelines were applied for the statistical analyses [27]. According to fall experience and anemic status, the subjects’ characteristics are reported as frequencies and percentages with Rao–Scott χ^2^ tests. The differences in nutrient intake, including energy, protein, iron, and vitamin C, are reported as frequencies and percentages with Rao–Scott χ^2^ tests. To calculate the strength of association, Phi (φ) values for the 2 × 2 table and Cramer’s V coefficient for the table bigger than 2 × 2 were computed [33]. According to anemia status, the nutrient intake is reported with mean and standard deviation with the Student’s *t*-test. The effect size was calculated with cohen’s d. The significant threshold (*p*) was set at 0.050. Univariate and multivariate binary logistic regression analyses were used to examine the associations between anemia and falls. In the first multivariate logistic regression analysis, anemia and age were input as independent variables. For binary multiple regression analysis, anemia, age, education, type of family, residential area, economic activity, household income, high-risk drinking, smoking, physical activity, chronic disease, and BMI were analyzed to find an association with falls. The Variance Inflation Factor (VIF) and the mean VIF were used to assess the multicollinearity of the independent variables in the multivariate logistic regression model. All statistical analyses were performed using SAS 9.4 (SAS Institute, Cary, NC, USA).

## 3. Results

Of the 6665 subjects, 274 had a fall and 596 had anemia (Table 1). The incidence of falls was high when the type of family was living alone (χ^2^ = 7.32, *p* = 0.007, Phi (φ) = 0.033) and when economic activity was inactive (χ^2^ = 3.92, *p* = 0.048, Phi (φ) = 0.024). The prevalence of anemia increased with age (χ^2^ = 161.42, *p* < 0.001, Cramer’s V = 0.270). The prevalence of anemia was high when the type of family was living alone (χ^2^ = 10.60, *p* = 0.001, Phi (φ) = 0.033), when the education level was low (χ^2^ = 29.55, *p* < 0.001, Cramer’s V = 0.094), when the residential area was rural (χ^2^ = 5.06, *p =* 0.025, Phi (φ) = 0.002), when economic activity was inactive (χ^2^ = 17.41, *p* < 0.001, Phi (φ) = 0.024), when income was lower than 50% (χ^2^ = 33.65, *p* < 0.001, Phi (φ) = 0.016), when no aerobic exercise was performed (χ^2^ = 10.89, *p* = 0.001, Phi (φ) = 0.007), and when the patient had a chronic disease (χ^2^ = 35.99, *p* < 0.001, Phi (φ) = 0.015).

Anemia status in nutrient intake according to the EER and RNI is presented in Table 2. If the energy intake did not meet the EER, the prevalence state of anemia was significantly higher. When protein, iron, and vitamin C did not meet the RNI, the anemia state was considerably higher. The phi (φ) values for energy, protein, iron, and vitamin C were 0.033, 0.046, 0.051, and 0.043, respectively.

Nutrient intake amounts by anemia status are presented in Table 3. In the case of anemia, the intake levels of energy, protein, iron, and vitamin C were significantly lower than in cases of no anemia. Cohen’s d values for energy, protein, iron, and vitamin C were 0.064, 0.046, 0.089, and 0.072, respectively.

The three types of models for analyzing the association between anemia and falls are presented in Table 4. In the unadjusted model (OR = 1.89, *p* = 0.002), the age-adjusted model (OR = 1.84, *p* = 0.003), and the model in which the characteristics of all subjects were corrected (OR = 1.88, *p* = 0.003), the results showed that anemia was significantly related to the occurrence of falls.

## 4. Discussion

As a result of this study, it was confirmed that falls in postmenopausal women were related to anemia. Although it has not been established that anemia is a predisposing factor for falls, this study presents an opportunity of understanding that anemia should be included in the management items for managing falls. Similar to our study results, there has been a case where the relationship between falls and anemia in the elderly in the community was confirmed [5,34]. In terms of hospital admissions, more elderly people experiencing falls had anemia than those who did not [35]. In the annual health examination dataset analysis, the hemoglobin level of subjects who experienced a fall was lower than that of those who did not experience a fall [36]. However, there is not always a statistically significant relationship between anemia and falls in the community population [35,36]. Elderly aged 65 to 88 years of age living in the community recorded weekly falls for 3 years. [5] and injurious falls were confirmed through insurance claim data on which injuries related to falls. [34]. In a previous study, subjects were asked about their experiences of falls in the past year, as in this study [20,22,36]. The recall of past one-year experiences of falls is a subjective measure that relies on memory, and when we study fall occurrence at the community level, we must bear in mind the uncertainty of subjective reporting. It is necessary to conduct a prospective study to confirm the causal element of the occurrence of anemia as a preceding factor for falls in the elderly community base.

Anemia management is important for the health management of postmenopausal women, and it is necessary to prepare various intervention strategies to prevent falls in relation to anemia management, including dietary intervention. In anemia-related nutritional deficiency, nutrients frequently mentioned are vitamin B12, folate, riboflavin, lipoplatin, copper, protein deficiency, and iron deficiency [37]. In this study, the relationship between the insufficient energy intake of iron, protein, and vitamin C and anemia was confirmed. In terms of inadequate nutrient intake, the results of a prospective cohort study were similar those of this study [6]. Although the strength of association between nutrient intake and anemia is weak to moderate [33], it is necessary to regularly check for anemia in postmenopausal women and evaluate dietary intake to determine the adequacy of energy and nutrient intake in relation to anemia [6]. Anemia was significantly related to age in this study, (Rao–Scott χ^2^ =161.42, *p* > 0.001) and the strength of association was strong (Cramer’s V = 0.270). In addition to nutritional imbalance, further studies should be conducted to identify factors contributing to an increase in anemia with increasing age in menopausal women.

As a result of analyzing the relationship between anemia and age and the occurrence of falls, age was significantly associated with falls. Still, the correlation with the occurrence of falls was not confirmed in the full model. Instead, in the full model, it was confirmed that the type of family was significantly related to the occurrence of falls. It was found that the incidence of falls was lower when living with someone, such as a spouse or children, compared to living alone. In these instances, the relationship between age and fall decreased; the implications of living with family showed a tendency similar to the results of previous studies [21].

The limitations of this study are as follows. Although we used a survey representative of Korea, it is difficult to confirm the cause and effect of anemia and falls when analyzing cross-sectional data. Second, since falls were measured as a subjective response to falls in the past year, it is necessary to examine falls’ relationship with anemia by closely measuring the frequency of occurrence or the treatment patterns of falls in the future. Third, in this study, the intake patterns of major nutrients were examined in the context of anemia and related factors. The relationship with vitamin B12, which is often mentioned in the literature, was not confirmed because guidelines for KNHANES data analysis are not provided. In the future, when confirming the relationship between dietary patterns and anemia, it is necessary to analyze vitamin B12. Nevertheless, this study suggests that to prevent fall-induced injuries during the aging process of menopausal women, future interventions should confirm the relationship between anemia and nutrient intake and between anemia and falls in menopausal women.

## 5. Conclusions

We found that postmenopausal women with anemia were more likely to fall than subjects without anemia. Additionally, anemia was confirmed to be related to insufficient energy intake, protein, iron, and vitamin C. For the health management of postmenopausal women, it is necessary to educate these subjects on sufficient nutrient intake, managing anemia, and preventing falls.

## Figures and Tables

**Table 1:** Fall experience and anemia according to the subjects’ characteristics.

Variables	Category	Total	Fall Experience				Anemia			
		Unweighted n (Total)	Unweighted n (Weighted %)	Rao-Scott χ^2^ (df)	*p*	Phi (φ)/ Cramer’s V	Unweighted n (Weighted %)	Rao-Scott χ^2^ (df)	*p*	Phi (φ)/ Cramer’s V
Age (year) (64.14 ± 9.07) *	>64	3573	120 (3.4)	6.25	0.100		194 (5.6)	161.42	<0.001	0.270
	65–69	1066	61 (5.2)	(3)			92 (9.1)	(3)		
	70–74	890	42 (4.4)				111 (12.6)			
	75–79	1136	51 (4.0)				199 (18.0)			
Education	≥College	815	24 (3.0)	1.38	0.501		47 (5.3)	29.55	<0.001	0.094
	High school	1591	59 (3.8)				95 (6.5)	(2)		
	≤Middle school	4259	191 (4.0)				454 (10.6)			
Type of family	Living alone	1271	75 (5.3)	7.32 (1)	0.007	0.033	151 (11.7)	10.60	0.001	0.033
	Living with someone	5394	199 (3.6)				445 (8.2)			
Residential area	Urban area	5173	214 (3.8)	0.02	0.900		442 (8.3)	5.06	0.025	0.002
	Rural area	1492	60 (3.9)				154 (10.8)			
Economic activity	Inactive	3768	178 (4.3)	3.92	0.048	0.024	398 (10.3)	17.41	<0.001	0.024
	Active	2897	96 (3.2)	(1)			198 (6.9)	(1)		
Household income	High	2862	98 (3.5)	1.68	0.194		187 (6.5)	33.65	<0.001	0.016
	Low	3803	176 (4.2)				409 (10.8)	(1)		
High-risk drinking	No	6510	267 (3.8)	0	0.989		589 (8.9)	3.65	0.056	
	Yes	155	7 (3.8)				7 (4.1)			
Present smoker	No	6456	265 (3.8)	0.39	0.530		581 (9.0)	1.32	0.250	
	Yes	209	9 (4.8)				15 (7.2)			
Aerobic physical activity	No	4179	177 (4.0)	0.37	0.543		420 (9.9)	10.89	0.001	0.007
	Yes	2486	97 (3.6)				176 (7.0)			
Having chronic disease	No	1858	68 (3.4)	1.44	0.231		89 (5.0)	35.99	<0.001	0.015
	Yes	4807	206 (4.1)				507 (10.4)	(1)		
BMI (kg/m^2^) (24.24 ± 3.31) *	<18.5	136	5 (2.6)	4.43	0.109		13 (9.9)	5.59	0.061	
	18.5–24.9	4068	157 (3.5)				390 (9.4)			
	≥25	2461	112 (4.5)				193 (7.5)			

Note: df = degrees of freedom; * = mean ± standard deviation.

**Table 2:** Anemia status according to estimated energy requirements (EER) and recommended nutrient intake (RNI).

Variables	Category	No Anemia	Anemia	Rao–Scott χ^2^ (df)	*p*	
		Unweighted n (Weighted %)	Unweighted n (Weighted %)			Phi (φ)
Energy	Meets EER standards	2566 (92.5)	214 (7.5)	7.13 (1)	0.008	0.033
	Below EER	3503 (90.3)	382 (9.7)			
Protein	Meets RNI standards	2974 (92.8)	230 (7.2)	13.93 (1)	<0.001	0.046
	Below RNI	3095 (89.7)	366 (10.3)			
Iron	Meets RNI standards	4699 (92.2)	411 (7.8)	17.34 (1)	<0.001	0.051
	Below RNI	1370 (88.0)	185 (12.0)			
Vitamin C	Meets RNI standards	1940 (93.4)	133 (6.6)	12.17 (1)	0.001	0.043
	Below RNI	4129 (90.2)	463 (9.8)			

Note: df = degrees of freedom.

**Table 3:** Energy and nutrient intake amount by anemic status.

Energy and Nutrient Intake	No Anemia	Anemia	*t*	*p*	Cohen’s *d*
	Mean ± SD	Mean ± SD			
Energy (kcal/day)	1598.16 ± 9.80	1518.54 ± 28.21	2.63	0.009	0.064
Protein (g/day)	53.71 ± 0.42	51.12 ± 1.32	1.88	0.060	0.046
Iron (g/day)	13.54 ± 0.17	12.20 ± 0.33	3.63	<0.001	0.089
Vitamin C (mg/day)	98.09 ± 2.38	81.42 ± 5.47	2.93	0.004	0.072

**Table 4:** Factors related to fall experience.

Model	Reference Category	Category	OR (95% CI)	*p*
Unadjusted	Anemia (Ref: no)	Yes	1.89 (1.27~2.81)	0.002
Age-adjusted	Age (Ref: under 64)	65–69	1.52 (1.06~2.18)	0.025
		70–74	1.23 (0.79~1.90)	0.356
		75–79	1.07 (0.72~1.60)	0.729
	Anemia (Ref: no anemia)	Anemia	1.84 (1.23~2.77)	0.003
Fully adjusted	Age (Ref: under 64)	65–69	1.36 (0.91~2.02)	0.131
		70–74	1.02 (0.63~1.66)	0.934
		75–79	0.84 (0.52~1.37)	0.494
	Education (Ref: ≤middle school)	≥College	0.90 (0.51~1.58)	0.716
		High school	1.11 (0.74~1.67)	0.614
	Type of family (Ref: living alone)	Living with someone	0.69 (0.50~0.94)	0.020
	Residential area (Ref: urban area)	Rural area	1.01 (0.71~1.44)	0.938
	Economic activity (Ref: inactive)	Active	0.78 (0.56~1.08)	0.134
	Household income (Ref: high)	Low	1.01 (0.71~1.44)	0.954
	High-risk drinking (Ref: no)	Yes	0.98 (0.40~2.43)	0.971
	Present smoking (Ref: no)	Yes	1.24 (0.53~2.92)	0.615
	Aerobic physical activity (Ref: no)	Yes	0.96 (0.70~1.32)	0.815
	Having chronic disease (Ref: no)	Yes	1.03 (0.70~1.52)	0.880
	BMI (Ref: 18.5–24.9)	<18.5	0.72 (0.28~1.83)	0.483
		≥25	1.28 (0.95~1.73)	0.109
	Anemia (Ref: no anemia)	Anemia	1.88 (1.24~2.84)	0.003

Note: OR = odds ratio.

## Data Availability

The data analyzed in this study are publicly open data. Interested parties can apply on the website to obtain the data.

## References

[1] de Benoist B., McLean E., Egli I., Cogswell M., World Health Organization (2008). Worldwide Prevalence of Anaemia 1993–2005: WHO Global Database on Anaemia.

[2] Chueh H.W., Jung H.L., Shim Y.J., Choi H.S., Han J.Y. (2020). High anemia prevalence in Korean older adults, an advent healthcare problem: 2007–2016 KNHANES. BMC Geriatr..

[3] Guralnik J.M., Eisenstaedt R.S., Ferrucci L., Klein H.G., Woodman R.C. (2004). Prevalence of anemia in persons 65 years and older in the United States: Evidence for a high rate of unexplained anemia. Blood.

[4] Tettamanti M., Lucca U., Gandini F., Recchia A., Mosconi P., Apolone G., Nobili A., Tallone M.V., DeToma P., Giacomin A. (2010). Prevalence, incidence and types of mild anemia in the elderly: The “Health and Anemia” population-based study. Haematologica.

[5] Penninx B.W.J.H., Pluijm S.M.F., Lips P., Woodman R., Miedema K., Guralnik J.M., Deeg D.J.H. (2005). Late-Life Anemia Is Associated with Increased Risk of Recurrent Falls. J. Am. Geriatr. Soc..

[6] Elsevier Health Sciences (2011). Inadequate Diet Can Lead to Anemia in Postmenopausal Women—ScienceDaily.

[7] Duman T.T., Aktas G., Atak B.M., Kocak M.Z., Kurtkulagi O., Bilgin S. (2019). General characteristics of anemia in postmenopausal women and elderly men. Aging Male.

[8] Fishman S.M., Christian P., West K.P. (2000). The role of vitamins in the prevention and control of anaemia. Public Health Nutr..

[9] Goetz L.G., Valeggia C. (2017). The ecology of anemia: Anemia prevalence and correlated factors in adult indigenous women in Argentina. Am. J. Hum. Biol..

[10] Thomson C.A., Stanaway J.D., Neuhouser M.L., Snetselaar L.G., Stefanick M.L., Arendell L., Chen Z. (2011). Nutrient Intake and Anemia Risk in the Women’s Health Initiative Observational Study. J. Am. Diet. Assoc..

[11] Minkin M.J. (2019). Menopause: Hormones, Lifestyle, and Optimizing Aging. Obstet. Gynecol. Clin. N. Am..

[12] El Khoudary S.R., Greendale G., Crawford S.L., Avis N.E., Brooks M.M., Thurston R.C., Waetjen L.E., Matthews K. (2019). The menopause transition and women’s health at midlife: A progress report from the Study of Women’s Health across the Nation (SWAN). Menopause.

[13] Tussing-Humphreys L., Braunschweig C. (2011). Anemia in postmenopausal women: Dietary inadequacy or nondietary factors?. J. Am. Diet Assoc..

[14] Tsujioka T., Tohyama K. (2008). Prevalence of anemia in Japan. Nihon Rinsho.

[15] Cooper J.W., Burfield A.H. (2009). Medication interventions for fall prevention in the older adult. J. Am. Pharm. Assoc..

[16] Sekaran N.K., Choi H., Hayward R.A., Langa K.M. (2013). Fall Associated Difficulty with Activities of Daily Living (ADL) in Functionally Independent Older Adults Aged 65 to 69 in the United States: A Cohort Study. J. Am. Geriatr. Soc..

[17] Pin S., Spini D. (2016). Impact of falling on social participation and social support trajectories in a middle-aged and elderly European sample. SSM-Popul. Health.

[18] Kim K.J., Heo M. (2021). The Relationship between Falling and Quality of Life for The Elderly over 65: Using Korean community health survey. J. Korean Soc. Integr. Med..

[19] Lee Y.G., Kim S.C., Chang M., Nam E., Kim S.G., Cho S.I., Ryu D.H., Kam S., Choi B.Y., Kim M.J. (2018). Complications and Socioeconomic Costs Associated With Falls in the Elderly Population. Ann. Rehabil. Med..

[20] Noh J.-W., Kim K.-B., Lee J.H., Lee B.-H., Kwon Y.D., Lee S.H. (2017). The elderly and falls: Factors associated with quality of life A cross-sectional study using large-scale national data in Korea. Arch. Gerontol. Geriatr..

[21] Kim T., Choi S.D., Xiong S. (2020). Epidemiology of fall and its socioeconomic risk factors in community-dwelling Korean elderly. PLoS ONE.

[22] Lin C.-H., Liao K.-C., Pu S.-J., Chen Y.-C., Liu M.-S. (2011). Associated Factors for Falls among the Community-Dwelling Older People Assessed by Annual Geriatric Health Examinations. PLoS ONE.

[23] Lee Y.-Y., Chen C.-L., Lee I.-C., Lee I.-C., Chen N.-C. (2021). History of Falls, Dementia, Lower Education Levels, Mobility Limitations, and Aging Are Risk Factors for Falls among the Community-Dwelling Elderly: A Cohort Study. Int. J. Environ. Res. Public Health.

[24] Kim M., Chang M., Nam E., Kim S.G., Cho S.-I., Ryu D.H., Kam S., Choi B.Y., Kim M.J. (2020). Fall characteristics among elderly populations in urban and rural areas in Korea. Medicine.

[25] Tang S., Liu M., Yang T., Ye C., Gong Y., Yao L., Xu Y., Bai Y. (2022). Association between falls in elderly and the number of chronic diseases and health-related behaviors based on CHARLS 2018: Health status as a mediating variable. BMC Geriatr..

[26] Ogliari G., Ryg J., Andersen-Ranberg K., Scheel-Hincke L.L., Masud T. (2021). Association between body mass index and falls in community-dwelling men and women: A prospective, multinational study in the Survey of Health, Ageing and Retirement in Europe (SHARE). Eur. Geriatr. Med..

[27] Korea Disease Control and Prevention Agency (2022). National Health and Nutrition Survey Raw Data Usage Guidelines 8th 1st and 2nd Years (2019–2020).

[28] Go Y.J., Lee D.C., Lee H.J. (2021). Association between handgrip strength asymmetry and falls in elderly Koreans: A nationwide population-based cross-sectional study. Arch. Gerontol. Geriatr..

[29] Kweon S., Kim Y., Jang M.-J., Kim Y., Kim K., Choi S., Chun C., Khang Y.-H., Oh K. (2014). Data Resource Profile: The Korea National Health and Nutrition Examination Survey (KNHANES). Int. J. Epidemiol..

[30] Oh K., Kim Y., Kweon S., Kim S., Yun S., Park S., Lee Y.-K., Kim Y.-T., Park O., Jeong E.K. (2021). Korea National Health and Nutrition Examination Survey, 20th anniversary: Accomplishments and future directions. Epidemiol. Health.

[31] Yun S.H., Shim J.-S., Kweon S., Oh K. (2013). Development of a Food Frequency Questionnaire for the Korea National Health and Nutrition Examination Survey: Data from the Fourth Korea National Health and Nutrition Examination Survey (KNHANES IV). Korean J. Nutr..

[32] The Korean Nutrition Society (2020). 2020 Dietary Reference Intakes for Koreans: Energy and Macronutrients.

[33] Akoglu H. (2018). User’s guide to correlation coefficients. Turk. J. Emerg. Med..

[34] Duh M.S., Mody S.H., Lefebvre P., Woodman R.C., Buteau S., Piech C.T. (2008). Anaemia and the Risk of Injurious Falls in a Community-Dwelling Elderly Population. Drugs Aging.

[35] Dharmarajan T., Avula S., Norkus E.P. (2007). Anemia Increases Risk for Falls in Hospitalized Older Adults: An Evaluation of Falls in 362 Hospitalized, Ambulatory, Long-Term Care, and Community Patients. J. Am. Med Dir. Assoc..

[36] Hopstock L.A., Utne E.B., Horsch A., Skjelbakken T. (2017). The association between anemia and falls in community-living women and men aged 65 years and older from the fifth Tromsø Study 2001–2002: A replication study. BMC Geriatr..

[37] Sanford A.M., Morley J.E. (2019). Editorial: Anemia of Old Age. J. Nutr. Health Aging.

